# The impact of record source on multimorbidity measurement and mortality associations: A comparison study

**DOI:** 10.23889/ijpds.v8i2.2191

**Published:** 2023-09-14

**Authors:** Clare MacRae

**Affiliations:** 1 University of Edinburgh, Edinburgh, United Kingdom

## Objectives

Measurement of multimorbidity is important; the impact of choice of record source on multimorbidity measurement is uncertain. We compared estimated prevalence of multimorbidity and associations with mortality using different record sources.

## Method

Cross-sectional study of SAIL Databank data including 2,340,027 individuals of all ages living in Wales on 01 January 2019. Comparison of multimorbidity and LTC prevalence using diagnosis codes from primary care (PC), hospital inpatient (HI), and linked PC-HI records, and associations with 12-month mortality were calculated.

## Results

Multimorbidity was more prevalent (32.2% versus 16.4%), included a younger population (62.5 years versus 66.8 years) with more women (54.2% versus 52.6%), and was more strongly associated with mortality (adjusted odds ratio 7.93 [95%CI 7.62-8.25] versus 4.70 [95%CI 4.55-4.85] in people with ≥ 4 LTCs) using linked PC-HI than HI records. Prevalence of LTCs using PC versus HI records was significantly higher in 37/47 and significantly lower in 10/47. Averaging all LTCs, the ratio of PC/linked PC-HI records was higher than HI/linked PC-HI records (84.7% [interquartile range, IQR 76.7 to 90.4] versus 49.0 [IQR 31.9 to 83.6]). Measures of concordance between PC and HI records varied widely across LTCs and the Kappa statistic, a comparison of percentage agreement and chance alone, was slight in five, fair in 13, moderate in 17, and substantial in 12 LTCs, and lowest in mental and behavioural disorders.

## Conclusion

Using linked PC-HI records provides higher estimated prevalence of multimorbidity with higher proportions of certain populations and higher association with mortality. Use of single record sources will underestimate the prevalence of many important conditions, especially mental and behavioural disorders.

